# Characteristics and Correlations of the Oral and Gut Fungal Microbiome with Hypertension

**DOI:** 10.1128/spectrum.01956-22

**Published:** 2022-12-08

**Authors:** Bo-Yan Chen, Wen-Zhen Lin, Yu-Lin Li, Chao Bi, Lin-Juan Du, Yuan Liu, Lu-Jun Zhou, Ting Liu, Shuo Xu, Chao-Ji Shi, Hong Zhu, Yong-Li Wang, Jian-Yong Sun, Yan Liu, Wu-Chang Zhang, Zhiyuan Zhang, Hui-li Zhang, Ya-Qin Zhu, Sheng-Zhong Duan

**Affiliations:** a Laboratory of Oral Microbiota and Systemic Diseases, Shanghai Ninth People’s Hospital, College of Stomatology, Shanghai Jiao Tong University School of Medicine, Shanghai, China; b National Center for Stomatology, Shanghai Key Laboratory of Stomatology, Shanghai, China; c National Clinical Research Center for Oral Diseases, Shanghai Key Laboratory of Stomatology, Shanghai, China; d Department of General Dentistry, Shanghai Ninth People’s Hospital, Shanghai Jiao Tong University School of Medicine, Shanghai, China; e Department of Stomatology, First Affiliated Hospital, Anhui Medical University, Hefei, China; f Department of Oral and Maxillofacial-Head and Neck Oncology, Shanghai Ninth People’s Hospital, Shanghai Jiao Tong University School of Medicine, Shanghai, China; g Department of Cardiology, Shanghai Ninth People’s Hospital, Shanghai Jiao Tong University School of Medicine, Shanghai, China; Nanchang University

**Keywords:** gut fungal microbiome, hypertension, oral fungal microbiome, oral-gut fungal correlations

## Abstract

The mycobiome is an essential constituent of the human microbiome and is associated with various diseases. However, the role of oral and gut fungi in hypertension (HTN) remains largely unexplored. In this study, saliva, subgingival plaques, and feces were collected from 36 participants with HTN and 24 healthy controls for metagenomic sequencing. The obtained sequences were analyzed using the Kraken2 taxonomic annotation pipeline to assess fungal composition and diversity. Correlations between oral and gut fungi and clinic parameters, between fungi within the same sample types, and between different sample types were identified by Spearman’s correlation analysis. Overall, the subgingival fungal microbiome had substantially higher alpha diversity than the salivary and fecal fungal microbiomes. The fungal microbiomes of the three sample types displayed distinct beta diversity from each other. Oral fungi but not gut fungi in HTN had beta diversity significantly different from that of controls. Among the fungi shared in the oral cavity and gut, *Exophiala* was the genus with the most notable changes. Exophiala spinifera was the most abundant salivary species in HTN. Some fungal species directly correlated with blood pressure, including gut Exophiala xenobiotica and Exophiala mesophila. The markedly impaired ecological cocorrelation networks of oral and gut fungi in HTN suggested compromised association among fungal species. Most fungi were shared in the oral cavity and gut, and their correlations suggested the potential interplays between oral and gut fungi. In conclusion, the oral cavity and intestine have unique fungal ecological environments. The fungal enrichment and ecology in HTN, the correlations between oral and gut fungi, and the associations between oral and gut fungi and clinical parameters suggest an important role that the fungal microbiome may play in HTN.

**IMPORTANCE** Our study fills the gap in human studies investigating the oral and gut fungal microbiota in association with blood pressure. It characterizes the diversity and composition of the oral and gut fungal microbiome in human subjects, elucidates the dysbiosis of fungal ecology in a hypertensive population, and establishes oral-gut fungal correlations and fungus-clinical parameter correlations. Targeting fungi in the oral cavity and/or gut may provide novel strategies for the prevention and treatment of hypertension.

## INTRODUCTION

Hypertension (HTN) is a chronic disease of persistently elevated blood pressure (BP) that has deleterious effects on various other diseases. HTN increases the risk of cardiovascular diseases, chronic kidney disease, and dementia, as well as all-cause mortality worldwide (1–4). Despite tremendous efforts for BP control, the prevalence of HTN remains high (5, 6). Better understanding of the pathophysiological basis of HTN is essential for successful prevention and treatment of this disease.

Recent studies, mostly focusing on bacterial microbiotas, have illustrated that the microbiota and its interactions with the host play essential roles in HTN. Emerging evidence has suggested solid relationships between the bacterial microbiome and HTN (4, 7). For example, transplantation of fecal microbiotas of HTN patients in germfree mice significantly increases the BP of the recipient hosts (8). For another example, oral commensal bacteria are essential for the conversion from nitrate to nitrite (and ultimately nitric oxide), an important mechanism mediating the BP-lowering benefit of dietary nitrates (9). The research progress on fungal communities has been much slower than that on bacteria because of the lower abundance (10) and the lack of a well-defined reference genomic database (11). Nonetheless, a few studies have started to reveal the important roles of the fungal microbiota in human health and disease (12, 13). For example, recent studies have demonstrated that the distinct gut fungal microbiome is associated with various diseases, including inflammatory bowel diseases (14), colorectal cancer (15), liver cirrhosis (16), multiple sclerosis (17), and Rett syndrome (18).

However, very few studies have investigated the function of fungi in HTN. *Monascus*, an edible fungus, is a traditional Chinese medicine that has been used for several centuries, and its fermented products have been reported to have antihypertensive benefits (19). Medicinal substances derived from the nonpathogenic fungi Aspergillus oryzae and Aspergillus luchuensis also exert antihypertensive effects (20). Thus, characterization of the fungal microbiome in HTN patients is critical to understanding the potential roles and mechanisms of fungi in the development and progression of HTN.

This study aimed to characterize the oral and gut fungal microbiome in HTN by metagenome sequencing. First, we analyzed the oral and gut fungal profiles in diversity and composition and compared HTN patients with participants without HTN (no HTN). Then, we compared the fungal enrichment between no-HTN and HTN participants. Subsequently, we studied correlations between oral and gut fungal species and BP and other clinical parameters and determined possible HTN-specific impairments of fungal interactions. Finally, we investigated correlations between oral and gut fungal species in HTN.

## RESULTS

### Diversity and composition of the fungal microbiomes in saliva, subgingival plaques, and feces.

**FIG 1:**
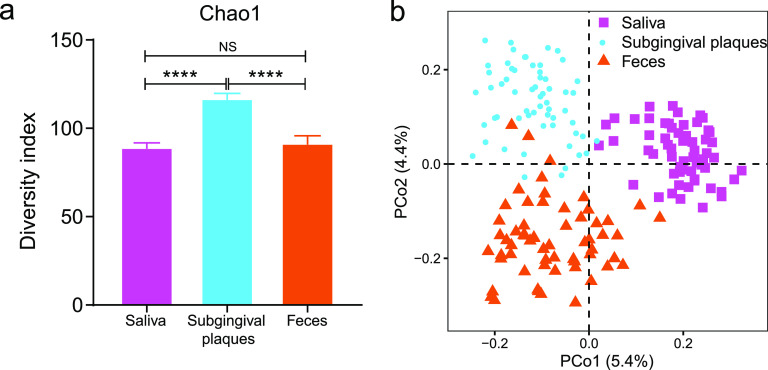
The fungal diversity of oral and fecal samples in the study population. (a) Chao1 alpha diversity indices of fungi in saliva, subgingival plaques, and feces. (b) Beta diversity at the species level assessed by principal-coordinate analysis (PCoA) of Bray-Curtis distance. Student’s *t* test was used for statistical analysis in panel a. *n* = 60 for all sample types. NS, no significance. ****, *P* < 0.0001.

To investigate the potential function of fungi in hypertension, we performed shotgun metagenomic sequencing of oral (saliva and subgingival plaques) and gut (feces) samples taken from 24 HTN participants and 36 no-HTN participants. The alpha diversity of the fungal microbiome was substantially higher in subgingival plaques than in saliva and feces as measured by Chao1, Shannon, and ACE diversity indexes (Fig. 1a; see also Fig. S1 in the supplemental material). The salivary and gut fungal microbiomes had similar alpha diversities. Principal-coordinate analysis (PCoA) for the beta diversity revealed three distinctly separate clusters corresponding to saliva, subgingival plaques, and feces, demonstrating different fungal compositions among these three sample types, even though saliva and subgingival plaques are both oral samples (Fig. 1b).

The predominant (relative abundance > 10%) fungi were *Ascomycota* and *Basidiomycota* at the phylum level, *Saccharomycetes* at the class level, and *Saccharomycetales* at the order level in both oral and gut samples (Fig. S2). The predominant families were *Saccharomycetaceae* in saliva and feces and *Debaryomycetaceae* in subgingival plaques. *Candida* and *Saccharomyces* were the most abundant genera in oral and gut samples, respectively. The relative abundances of the species Candida albicans, Saccharomyces cerevisiae, and Malassezia globosa were comparable in saliva (Fig. S2a). Candida albicans and Saccharomyces cerevisiae were the most abundant species in subgingival plaques and feces, respectively (Fig. S2b and c). The *Malasseziomycetes* class and *Malasseziales* order in saliva, the *Leotiomycetes* class and *Polyporales* order in feces, and the *Pleosporales* order in subgingival plaques were more abundant than the corresponding taxa in the other two sample types (Fig. S2a to c). These results suggested that the overall fungal composition and abundances between the oral and gut samples varied.

### Comparison of the oral and gut fungal diversities and compositions between no-HTN and HTN patients.

**FIG 2:**
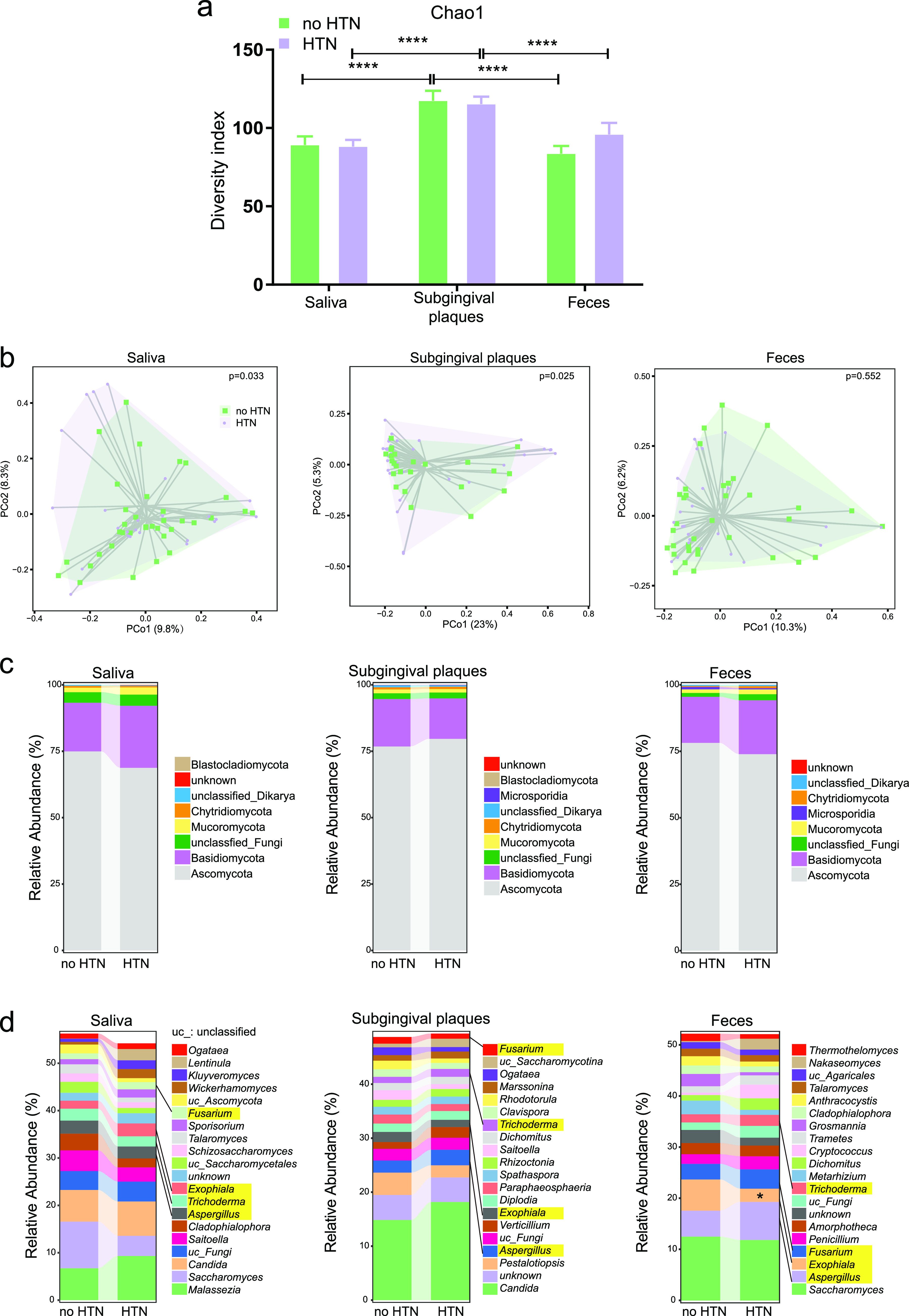
Comparison of the oral and gut fungal diversity and composition between the no-HTN and HTN groups. (a) Chao1 alpha diversity indices of fungi in saliva, subgingival plaques, and feces of the no-HTN and HTN groups. (b) Beta diversity at the species level assessed by PCoA of Bray-Curtis distance in saliva, subgingival plaques, and feces of the no-HTN and HTN groups. (c and d) Fungal composition in saliva, subgingival plaques, and feces of the no-HTN and HTN groups at the phylum (c) and genus (d) levels. The top 20 genera are depicted in panel d. The four genera shared by all three sample types are highlighted in yellow. Student’s *t* test was used for statistical analysis in panels a and d, and permutational multivariate analysis of variance was used for panel b. *n* = 24:36 (no HTN:HTN) for all sample types. *, *P* < 0.05; ****, *P* < 0.0001.

We next investigated the differences in fungal diversity and composition between the no-HTN and HTN groups. The Chao1 alpha diversities of the fungal microbiomes were similar between the no-HTN and HTN groups for saliva and subgingival plaques (*P* > 0.1), while that of the HTN group tended to be higher than that of the no-HTN group for feces (Fig. 2a) (*P* = 0.09). Consistent with the results of the overall fungal community, the alpha diversity (Chao1, Shannon, and ACE) was higher in subgingival plaques than in saliva and feces for both the no-HTN and HTN groups (Fig. 2a and Fig. S3). PCoA demonstrated that the beta diversities of both the salivary and subgingival fungal microbiomes were significantly different between the no-HTN and HTN groups, but those of the fecal fungal microbiomes were indistinguishable between the two groups (Fig. 2b). The relative abundances of the predominant oral and gut fungal microbiomes differed between the no-HTN and HTN groups (Fig. 2c). At the phylum level, *Ascomycota* was decreased in saliva and feces but increased in subgingival plaques of the HTN group. Conversely, *Basidiomycota* was decreased in subgingival plaques but increased in saliva and feces of the HTN group. The most abundant oral genera, including salivary *Malassezia* and subgingival *Candida*, were increased in the HTN group, and the most abundant gut genus, *Saccharomyces*, was decreased in the HTN group (Fig. 2d). Among the four shared genera (Fusarium, *Exophiala*, *Trichoderma*, and Aspergillus, highlighted in yellow in Fig. 2d) in the three sample types, *Exophiala* had the most apparent changes between the no-HTN and HTN groups.

**FIG 3:**
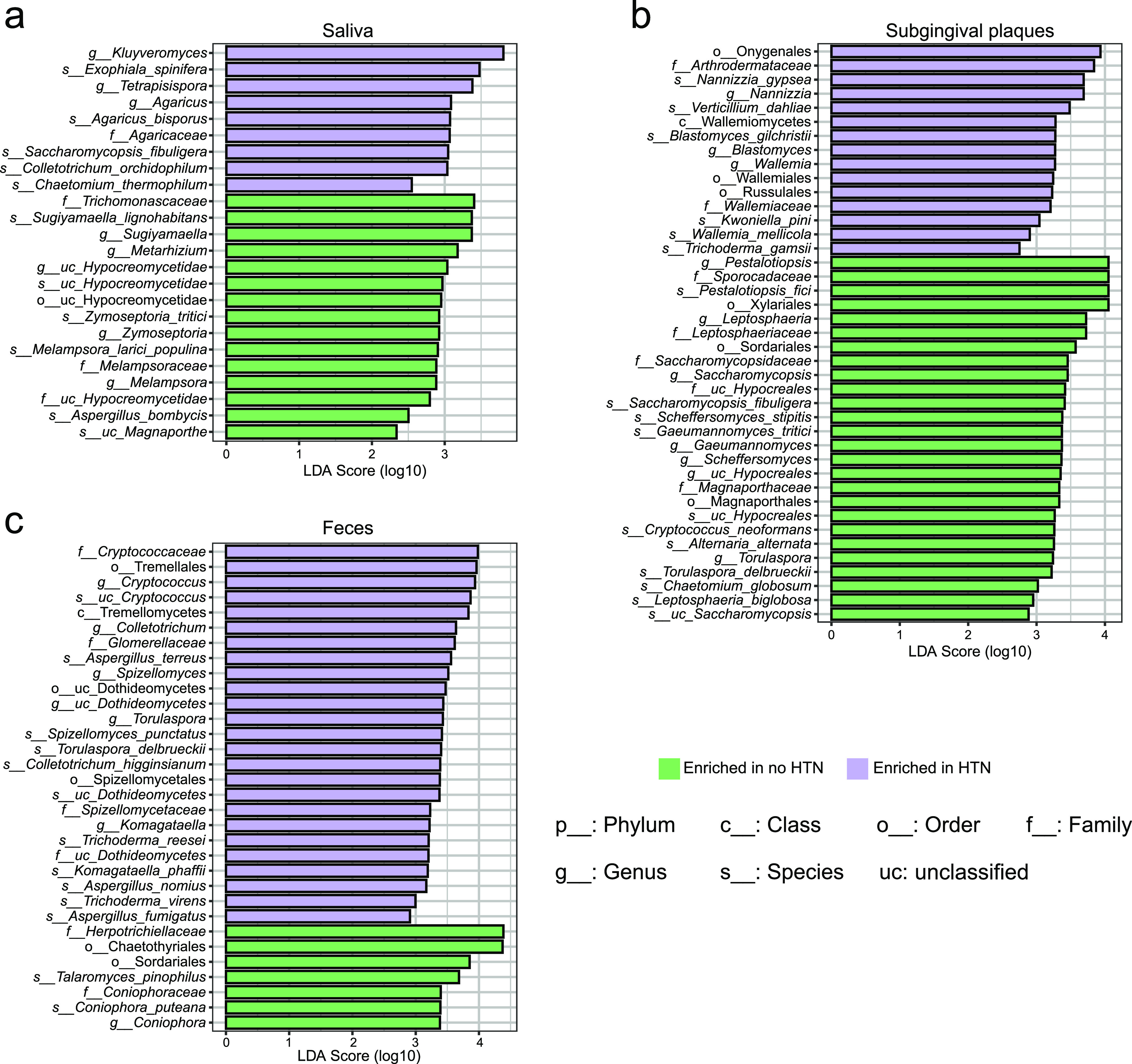
Different fungal enrichments between the no-HTN and HTN groups. Shown is linear discriminant analysis (LDA) effect size (LEfSe) analysis of fungal enrichment in saliva (a), subgingival plaques (b), and feces (c) of the no-HTN and HTN groups. The threshold LDA score was 2. *n* = 24:36 (no HTN:HTN) for all sample types.

Linear discriminant analysis (LDA) effect size was used to further detect the difference in fungal enrichment between the no-HTN and HTN groups. The enrichment levels and their hierarchical relationships are shown by bar charts of LDA scores and cladograms (Fig. 3 and Fig. S4). The most enriched families in the saliva of the HTN and no-HTN groups were *Agaricaceae* and *Trichomonascaceae*, respectively (Fig. 3a). The most enriched class in the subgingival plaques of the HTN group was *Wallemiomycetes*, and most enriched order of the no-HTN group was *Xylariales* (Fig. 3b). The most enriched class in the gut of the HTN group and the most enriched order of the no-HTN group were *Tremellomycetes* and *Chaetothyriales*, respectively (Fig. 3c). The enrichment of these fungal taxa included their subordinate genera such as salivary *Agaricus*, subgingival *Wallemia*, and gut Cryptococcus in the HTN group and salivary *Sugiyamaella* and subgingival *Pestalotiopsis* in the no-HTN group (Fig. S4a to c). The most enriched species in the HTN group were Exophiala spinifera in saliva, Nannizzia gypsea in subgingival plaques, and an unclassified taxon under Cryptococcus in feces (Fig. 3a to c); those in the no-HTN group were Sugiyamaella lignohabitans, Pestalotiopsis fici, and Talaromyces pinophilus in saliva, subgingival plaques, and feces, respectively. Interestingly, consistent with the changes of the shared taxa in oral cavity and gut at the genus level (Fig. 2d), corresponding subordinate species such as salivary *Exophiala spinifera*, subgingival Trichoderma gamsii, and fecal Aspergillus terreus, Aspergillus nomius, Aspergillus fumigatus, Trichoderma reesei, and Trichoderma virens all enriched in the HTN group (Fig. 3a to c). These data revealed notable alterations of the human fungal microbiome in the HTN group.

### Associations between the oral and gut fungal microbiomes and clinical parameters.

**FIG 4:**
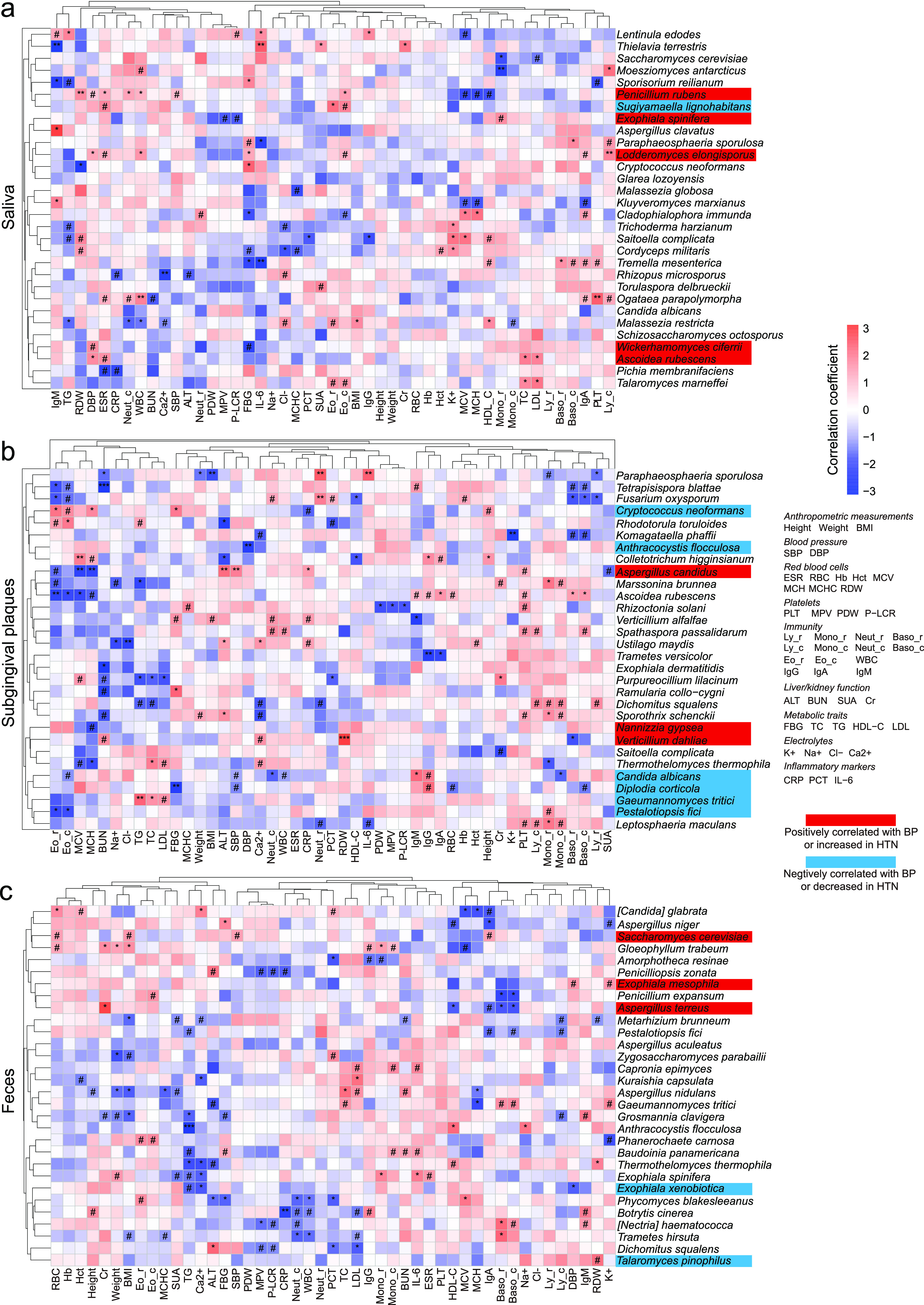
Correlations between oral and gut fungi and blood pressure and other clinical parameters. Shown are heat maps of Spearman’s correlation coefficients between clinical parameters and relative abundances of the top 30 fungal species in saliva (a), subgingival plaques (b), and feces (c). BMI, body mass index; SBP, systolic blood pressure; DBP, diastolic blood pressure; ESR, erythrocyte sedimentation rate; RBC, red blood cells; Hb, hemoglobin; Hct, hematocrit; MCV, mean corpuscular volume; MCH, mean corpuscular hemoglobin; MCHC, mean corpuscular hemoglobin concentration; RDW, red blood cell distribution width; PLT, platelet; MPV, mean platelet volume; PDW, platelet distribution width; P-LCR, platelet-large cell ratio; Ly_ r, lymphocyte ratio; Mono_r, monocyte ratio; Neut_ r, neutrophil ratio; Eo_r, eosinophil ratio; Baso_ r, basophil ratio; Ly_c, lymphocyte count; Mono_c, monocyte count; Neut_c, neutrophil count; Baso_c, basophil count; Eo_c, eosinophil count; WBC, white blood cells; Ig, immunoglobulin; ALT, alanine aminotransferase; BUN, blood urea nitrogen; SUA, serum uric acid; Cr, creatinine; FBG, fasting blood glucose; TC, total cholesterol; TG, triglyceride; HDL-C, high-density lipoprotein cholesterol; LDL, low-density lipoprotein cholesterol; K+, potassium; Na+, sodium; Cl−, chlorine; Ca2+, calcium; CRP, C-reactive protein; PCT, procalcitonin; IL-6, interleukin-6. *n* = 60 for all sample types. #, *P* (false-discovery rate [FDR]) < 0.1; *, *P* (FDR) < 0.05; **, *P* (FDR) < 0.01; ***, *P* (FDR) < 0.001.

We further analyzed associations between clinical parameters and the fungal microbiome. Among the top 30 species in oral and gut samples, there were 27 species in saliva, 30 species in subgingival plaques, and 29 species in feces significantly associated with various clinical parameters (Fig. 4a to c). We identified 115 significant correlations between the fungal microbiome and clinical parameters in saliva, 155 in subgingival plaques, and 139 in feces. There were 11 species that significantly correlated with systolic blood pressure (SBP) or diastolic blood pressure (DBP): Wickerhamomyces ciferrii, Ascoidea rubescens, Penicillium rubens, and Lodderomyces elongisporus in saliva, Aspergillus candidus in subgingival plaques, and Saccharomyces cerevisiae and Exophiala mesophila in feces positively correlated with BP (Fig. 4a to c, highlighted in red); Candida albicans, Diplodia corticola, and Anthracocystis flocculosa in subgingival plaques and Exophiala xenobiotica in feces negatively correlated with BP (Fig. 4a to c, highlighted in blue).

The fungal relative abundances had the highest number of significant correlations with immunity-related parameters (immunoglobulins and white blood cell count/ratio) among all clinical parameters. The salivary species *Lodderomyces elongisporus* positively correlated with immunoglobulin A and lymphocyte, eosinophil, and white blood cell counts; Ogataea parapolymorpha positively correlated with immunoglobulin A and lymphocyte, neutrophil, and white blood cell counts; and Malassezia restricta positively correlated with eosinophil ratio and negatively correlated with monocyte, neutrophil, and white blood cell counts (Fig. 4a). The subgingival species Fusarium oxysporum positively correlated with neutrophil count/ratio and negatively correlated with eosinophil and basophil count/ratio and lymphocyte ratio; *Ascoidea rubescens* positively correlated with immunoglobulins G, A, and M and basophil count/ratio and negatively correlated with eosinophil count/ratio (Fig. 4b). The fecal species Botrytis cinerea positively correlated with immunoglobulins G and M and negatively correlated with neutrophil and white blood cell counts; Nectria haematococca positively correlated with immunoglobulin M and basophil count/ratio and negatively correlated with neutrophil count (Fig. 4c).

Intriguingly, species that positively correlated with BP also positively correlated with immunity-related parameters or metabolic traits. For instance, salivary *Penicillium rubens* positively correlated with white blood cell, eosinophil, and neutrophil counts; salivary *Ascoidea rubescens* positively correlated with low-density lipoprotein cholesterol and triglyceride (Fig. 4a to c). Conversely, species that negatively correlated with BP also negatively correlated with immunity-related parameters or metabolic traits. For instance, subgingival *Diplodia corticola* negatively correlated with basophil count; gut *Exophiala xenobiotica* negatively correlated with triglyceride. Interestingly, besides the direct correlation between BP and gut *Exophiala mesophila*/*Exophiala xenobiotica*, other *Exophiala* spp. were also associated with HTN indirectly. For example, salivary and gut *Exophiala spinifera* positively correlated with monocyte ratio, and subgingival *Exophiala dermatitidis* negatively correlated with blood urea nitrogen. Both monocyte ratio and blood urea nitrogen were related to BP as previously reported (21, 22).

### Decreased fungal interactions in HTN.

**FIG 5:**
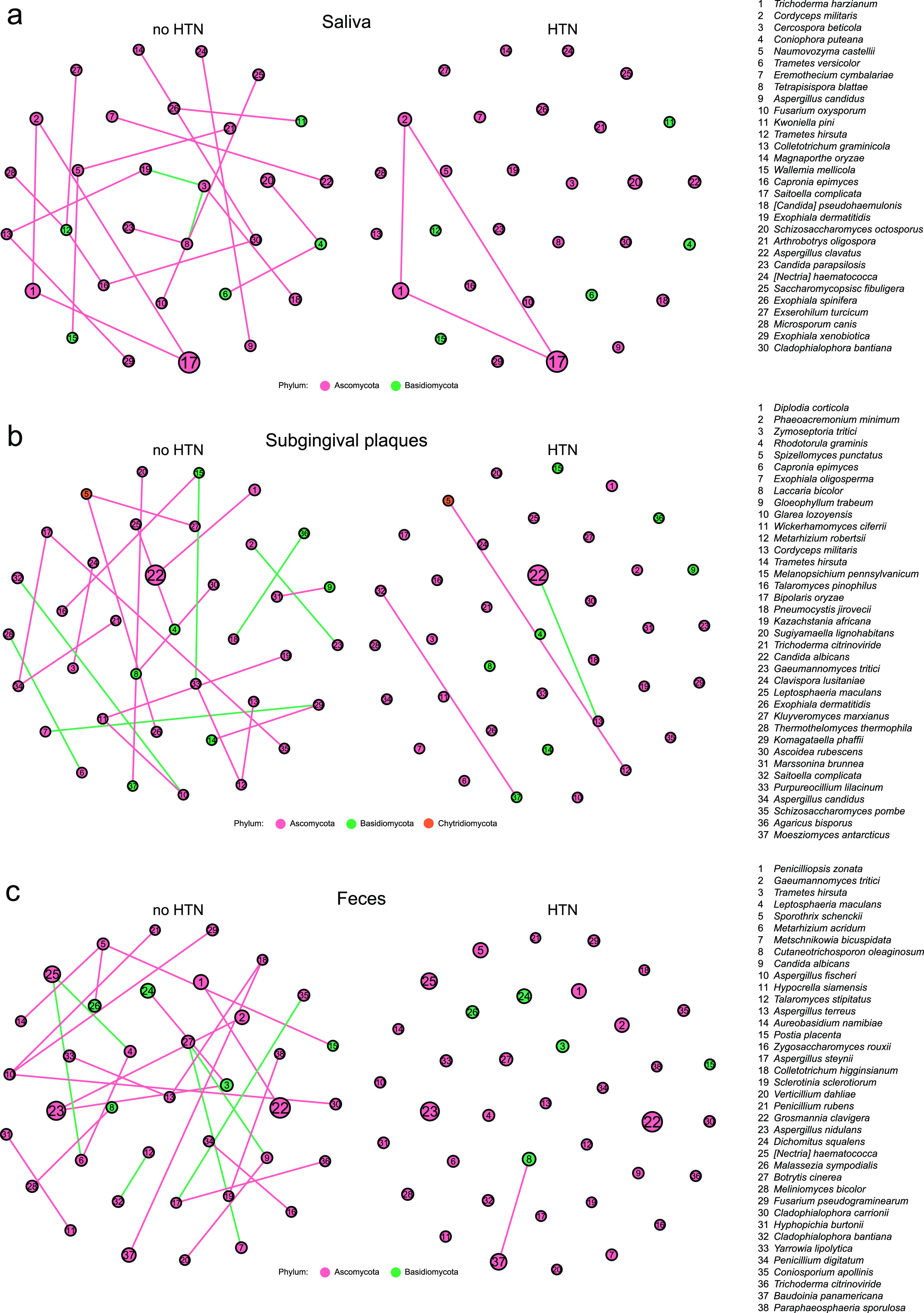
Different cocorrelation networks of predominant oral and gut fungal species between the no-HTN and HTN groups for saliva (a), subgingival plaques (b), and feces (c). The size of circles indicates the relative abundance of fungi. Pink and green lines indicate positive and negative correlations, respectively. The top 50 most abundant fungal species with *r* of >0.6 and *P* of <0.01 by Spearman’s correlation analysis are displayed. *n* = 24:36 (no HTN:HTN) for all sample types.

Microbial interactions play a vital role in maintaining host homeostasis (23). We generated fungal abundance cocorrelation networks to investigate the fungal co-occurrence and co-exclusion relationships and compared the differences between no-HTN and HTN participants. Overall, there were substantially more positive correlations than negative ones, suggesting that the fungal microbiomes of all three sample types had more co-occurrence than co-exclusion (Fig. 5a to c and Table S2). Moreover, the numbers of correlations (*r* > 0.6 and *P* < 0.01) among the top 50 most abundant fungal species were decreased dramatically in all three sample types of HTN participants compared to the no-HTN counterparts: from 22 to 3 in saliva, from 23 to 3 in subgingival plaques, and from 26 to 1 in feces. For instance, the correlations that oral *Exophiala* spp. (Exophiala dermatitidis, *Exophiala xenobiotica*, *Exophiala spinifera*, and Exophiala oligosperma) and gut Aspergillus spp. (Aspergillus fischeri, Aspergillus terreus, Aspergillus steynii, and Aspergillus nidulans) had with other species in the no-HTN group all disappeared in the HTN group. These results revealed that impaired interaction among fungal species was a prominent feature of the fungal microbiome in HTN.

### Correlations between the oral and gut fungal microbiomes in HTN.

The enrichment of gut-traveling oral bacteria has been linked to diseases (24, 25). It is not known whether oral fungi may travel to the gut and contribute to diseases such as HTN. To understand the potential oral-gut associations of fungi, we performed Spearman’s correlation analysis on fungal relative abundances between oral and gut samples.

**FIG 6:**
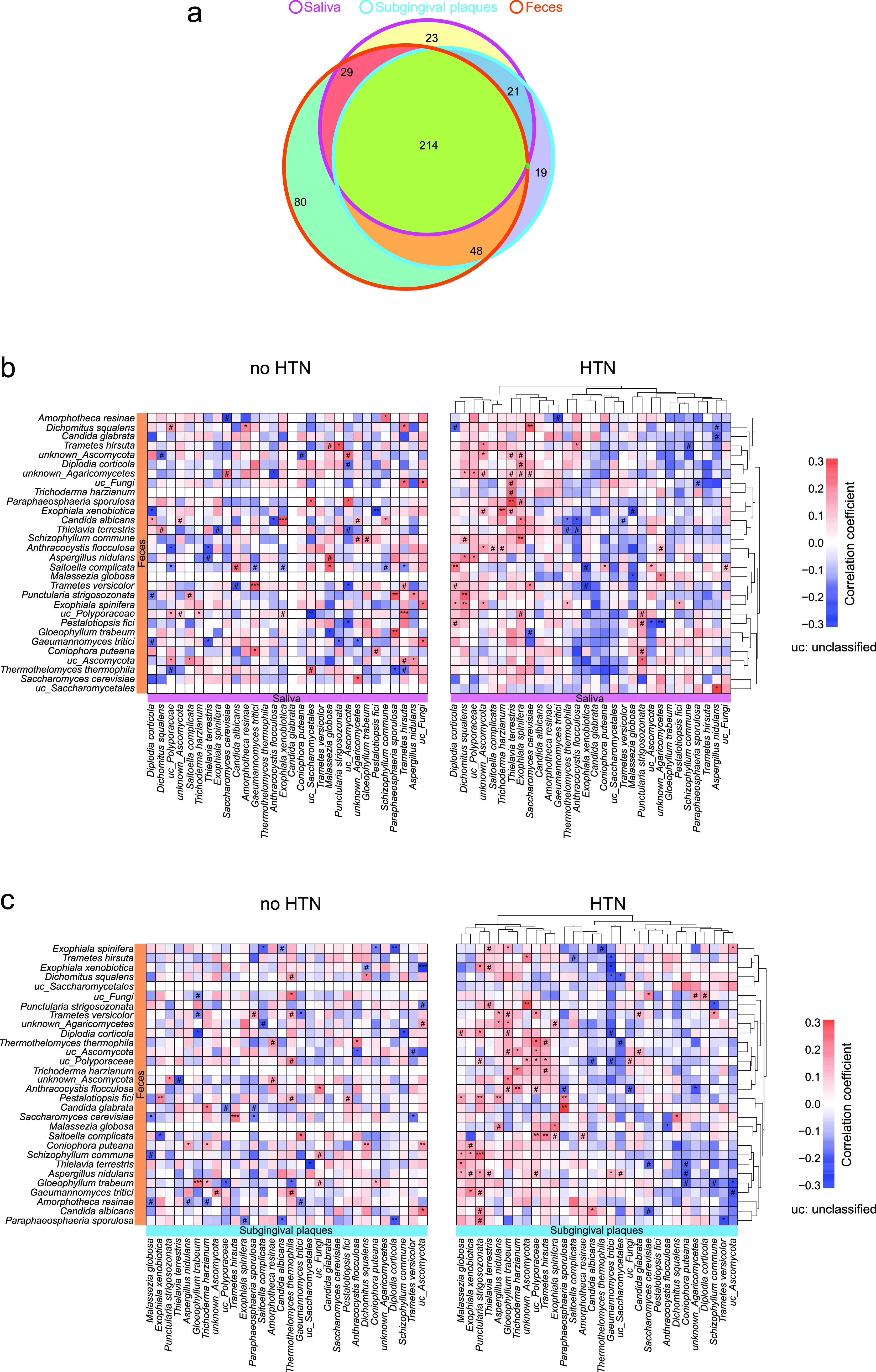
Associations between oral and gut fungal microbiota in the no-HTN and HTN groups. (a) Venn diagram of the shared and unique fungal species among saliva, subgingival plaques, and feces. The 30 most abundant fungal species out of the 214 shared species were used for analyses in panels b and c. (b and c) Heat maps of Spearman’s correlation coefficients between relative abundances of the top 30 shared fungal species in feces and those in saliva (b) or subgingival plaques (c). *n* = 60 for all sample types in panel a; *n* = 24:36 (no HTN:HTN) for all sample types in panels b and c. #, *P* (FDR) < 0.1; *, *P* (FDR) < 0.05; **, *P* (FDR) < 0.01; ***, *P* (FDR) < 0.001.

We identified 287 fungal species in saliva, 302 in subgingival plaques, 371 in feces, and 214 shared species in all three sample types (Fig. 6a). Among the top 30 shared species, *Exophiala xenobiotica* and *Anthracocystis flocculosa* were decreased and Candida albicans was increased in the HTN group compared to the no-HTN group in all sample types (Fig. S5 to S7). We identified 31 significant positive and 53 significant negative correlations between salivary and subgingival fungal species in no-HTN group and 63 significant positive and 26 significant negative correlations in the HTN group (Fig. S8). As for correlations between oral and gut fungi, there were 44 significant positive and 31 significant negative correlations in the no-HTN group and 49 significant positive and 18 significant negative correlations in the HTN group between saliva and feces (Fig. 6b). We also observed 30 significant positive and 35 significant negative correlations between subgingival and gut fungal species in the no-HTN group and 68 significant positive and 24 significant negative correlations in the HTN group (Fig. 6c).

We next explored correlations between the same fungal species in oral samples and in gut samples. None of the top 30 fungal species in saliva significantly correlated with the same species in the gut in the no-HTN group, and *Malassezia globosa* was the only salivary species that had significant correlation (negative) with the same fungus in gut in the HTN group (Fig. 6b). Subgingival Dichomitus squalens, *Pestalotiopsis fici*, and Gloeophyllum trabeum had significant positive correlations with the respective gut species in the no-HTN group, and Candida albicans and Trichoderma harzianum had significant positive correlations with the respective gut species in the HTN group (Fig. 6c).

Interestingly, salivary *Exophiala spinifera* and subgingival Punctularia strigosozonata in the HTN group had the largest numbers of correlations with gut fungi (Fig. 6b and c). Moreover, only *Exophiala spinifera* in both saliva and subgingival plaques had significant negative correlations with gut fungi in the no-HTN group and had significant positive correlations with gut fungi in the HTN group. In addition, oral *Exophiala spinifera* had significant positive correlations with gut *Malassezia globosa* and Candida albicans in the HTN group. Correlations between shared fungi within the oral and gut samples were also analyzed (Fig. S9a to c). It is worth noting that all significant correlations between the top 30 oral-gut shared fungal species within the gut were positive (Fig. S9c). These results together implied potential direct relationships between oral and gut fungal species, which might play an important role in HTN.

## DISCUSSION

The landscape of oral and gut fungal microbiomes in HTN is largely unknown. In this study, we performed metagenomic sequencing of saliva, subgingival plaques, and feces samples to investigate the fungal compositional and ecological differences among the three types of samples, and we compared the differences between the no-HTN and HTN groups within each type of samples. For the first time, our results elucidated the alterations of the fungal microbiome in HTN, established the associations between fungal species and clinical parameters, and revealed the correlations between oral and gut fungal species. This study provides an important reference for understanding the oral and gut fungi in HTN.

The overall diversity and composition of the oral and gut fungi were different among different sample types. We compared the fungal diversity of saliva, subgingival plaques, and feces. The higher diversity in subgingival plaques suggested that the periodontal space was a more suitable habitat for diverse fungi. The overall fungal compositions of these three types of samples were similar. For example, *Ascomycota* and *Basidiomycota* were the predominant phyla in the oral cavity and intestine, consistent with previous reports (26–28). Meanwhile, different habitats also had their unique fungal characteristics. For instance, *Malassezia*, a genus previously known to be a skin commensal (29, 30), was prevalent in saliva but not in subgingival plaques or feces in our study. As opportunistic pathogens, *Candida* organisms were frequently detected in human fungal infections (31). Although saliva and feces contained *Candida*, our results suggested that the subgingival plaques might be more suitable for the colonization of *Candida* organisms because of their higher relative abundance, especially Candida albicans. The largest order under *Dothideomycetes* was *Pleosporales*, which was reported to be associated with skin infection (32, 33) and also had a higher relative abundance in subgingival plaques than in saliva and feces in our results. Saccharomyces cerevisiae, a prominent fungal species in the human gut (26, 34), was more abundant in feces than in saliva and subgingival plaques in our study. In addition, the distinctive beta diversities of fungi in saliva, subgingival plaques, and feces indicated that the ecological properties of the fungal microbiome were sample type specific. These results suggested that the environment of habitats might affect the ecological structure and shape the unique mycobiome at different body sites.

Disease-specific signatures of oral and gut fungi in HTN were depicted by the different fungal enrichment and decreased fungal interactions. On one hand, we identified HTN-specific changes in fungal composition, manifested by the enrichment of 9 salivary fungal taxa,15 subgingival taxa, and 25 gut taxa in HTN. All these fungi were reported for HTN for the first time. The pathogenic role of some species in infectious diseases has been reported. For instance, *Exophiala spinifera* (35), *Nannizzia gypsea* (36), Blastomyces gilchristii (37), and Wallemia mellicola (38) were related to skin infections. Also, Torulaspora delbrueckii (39), Aspergillus terreus (40), Aspergillus nomius (41), and Aspergillus fumigatus (42) were capable of disrupting host immunity and recognized as opportunistic pathogens for pulmonary infections. Previous reports indicated that Agaricus bisporus and Saccharomycopsis fibuligera had beneficial effects on human health (43–45), but these species might have adverse effects on BP regulation because of their enrichment in HTN. The extracellular slime produced by *Exophiala spinifera* could be in the form of either capsule or exopolysaccharides and was the main virulence factor of this species (46). Biofilms consisting of exopolysaccharides could perpetuate infection, enhance inflammation, and cause tissue damage or death (47). Given the enrichment of *Exophiala spinifera* in saliva and feces of the HTN participants, we speculated that it could affect BP by extracellular slime. On the other hand, our analysis revealed complex fungal ecological networks in the no-HTN group for oral and gut samples, which were substantially impaired in the HTN group. For example, Aspergillus spp. were commonly detected in saliva, subgingival plaques, and feces, and their relative abundance in subgingival plaques positively correlated with BP; the correlations between Aspergillus spp. and other fungi disappeared in all three sample types of the HTN subjects. Interestingly, co-occurrence involving *Trichoderma harzianum* existed in both the no-HTN and HTN groups for saliva, reflecting the active interactions of *Trichoderma* spp. with other fungi as previously reported (48). We inferred that the altered fungal microbiome community in HTN might provide the fungi with an adverse condition for fungal interactions. Together, the enriched fungal taxa and the impaired ecological network in HTN might contribute to the pathogenesis of this disease.

The associations between oral and gut fungi and clinical parameters provided new insights into potential causality between the fungal microbiome and HTN. Our results showed strong associations between the relative abundances of oral and gut fungi and BP. Previous studies have shown that Candida albicans (an opportunistic pathogen) and Saccharomyces cerevisiae (a probiotic yeast) exert proinflammatory and anti-inflammatory roles, respectively, in inflammatory bowel disease (14, 49). However, our results indicated that subgingival Candida albicans (negatively correlated with BP) and gut Saccharomyces cerevisiae (positively correlated with BP) might be friend and foe for BP control, respectively. In addition, subgingival Candida albicans had negative correlations with the amounts of monocytes, neutrophils, and eosinophils, all of which have been adversely associated with the pathogenesis of HTN (21, 50–52), further suggesting that Candida albicans played a protective role in this disease. The potentially detrimental role of gut Saccharomyces cerevisiae in HTN was also suggested by the positive correlation between this species and immunoglobulin A, which has been previously shown to positively correlate with BP, and immunoglobulin A nephropathy was a major cause of secondary HTN of renal origin (53, 54). BP-associated fungal species such as salivary *Ascoidea rubescens* and gut *Exophiala xenobiotica* also correlated with blood metabolic traits. Given the vital role of immunity and blood lipids in HTN (51, 55), and the correlations we observed between BP-associated fungal species and indicators of immunity or metabolic traits, it was conceivable that fungi affected HTN development through alterations of immune cells and lipids. Taken together, our results established comprehensive correlations between fungal species and various physiological parameters, providing insights and references for further study of specific fungus-host interactions.

The fungal associations between the oral cavity and gut may be another factor affecting HTN. The oral-gut axis of the bacterial microbiome has been established in different diseases by various methods (56–59). However, few studies have investigated the connections between oral and gut fungi. We observed that most fungal species were present in all three sample types—saliva, subgingival plaques, and feces—providing a basis for oral-gut fungal associations. We identified the complex associations between the oral and gut fungi. For fungal correlations between different sample types, we observed a disease-specific pattern with a higher number of significant positive correlations in the HTN group than in the no-HTN group. The significant correlations of the same species between saliva and subgingival plaques and feces suggested potential oral-gut fungal associations. Intriguingly, oral *Exophiala spinifera* had significant correlations with these oral-gut-associated species, indicating that *Exophiala spinifera* might affect direct connections between oral and gut fungi. Therefore, these data suggested that oral-gut fungal connections might affect the development and progression of HTN.

### Conclusions.

Our work revealed the unique mycobiome environments of the oral cavity and intestine and unveiled the potential role of the fungal microbiome in HTN. The findings provide an essential reference for therapeutic and preventive interventions of HTN. For a deeper understanding of the roles of the fungal microbiome in the development of HTN, future studies are warranted to verify the function and mechanism of the disrupted fungal composition and ecology.

## MATERIALS AND METHODS

### Study cohorts.

A total of 36 participants with HTN and 24 controls were recruited from the Department of Cardiology at Shanghai Ninth People’s Hospital. Based on the criteria of 2018 ESC/ESH guidelines (5), participants with systolic blood pressure (SBP) of ≥140 mm Hg and/or diastolic blood pressure (DBP) of ≥90 mm Hg was diagnosed with HTN. BP was measured with an Omron electronic sphygmomanometer in a sitting position. Three readings were recorded at 5-min intervals, and the average was used as the final BP. Participants were excluded if they met any of the following conditions: (i) smoking, (ii) pregnancy, (iii) antibiotic or probiotic treatment or oral/gut surgeries in the previous 2 months, (iv) having fewer than eight natural teeth, and (v) suffering from peripheral artery disease, autoimmune disease, heart failure, renal failure, cancer, irritable bowel syndrome, inflammatory bowel disease, or recurrent aphthous oral ulcers. The acquirement of all clinical parameters followed standard procedures.

### Sample collection.

A total of 180 samples (60 saliva samples, 60 subgingival plaque samples, and 60 feces samples) were collected from the 60 participants. The sample metadata and all clinical parameters are presented in Table S1 in the supplemental material. The procedures of sample collection were consistent with our previous report (60). Briefly, participants were required to rinse their mouths and avoid eating and drinking for at least an hour before collection of oral samples. Saliva was collected and preserved in 50-mL sterile tubes (Corning, New York, NY, USA) with saliva DNA preservation solution (Huayueyang Biotech, Beijing, China). Hu-Friedy subgingival curettes were used to collect subgingival plaques, which were preserved in 2-mL sterile tubes (Eppendorf, Hamburg, Germany) with 20% glycerol. Fecal samples were freshly collected from each participant by providing collection containers in boxes with ice packs. All samples were transported to the laboratory on ice packs within 2 h and stored at −80°C. Whole-blood samples were collected in tubes with anticoagulants (Improve Medical, Guangzhou, China) after at least 8 h of fasting.

### Metagenome DNA extraction and shotgun sequencing.

The OMEGA kit (M5635-02; Omega Bio-Tek, Norcross, GA, USA) was used to extract microbial genomic DNA according to the manufacturer’s instructions. A NanoDrop NC2000 spectrophotometer (Thermo Fisher Scientific, Waltham, MA, USA) was used to measure the quantity of extracted DNA. The quality of extracted DNA was measured by agarose gel electrophoresis. The extracted microbial DNA was processed to construct metagenome shotgun sequencing libraries with insert sizes of 400 bp by using the Illumina TruSeq Nano DNA LT library preparation kit (Illumina, CA, USA). Each library was sequenced by the Illumina NovaSeq platform with PE150 (Paired End, 150bp) strategy at Personal Biotechnology Co., Ltd. (Shanghai, China).

### Bioinformatic analysis.

After sequencing, raw sequence data were filtered for further analysis. First, Cutadapt (v1.2.1) was used to remove adapters by identifying and cutting potential adapter sequences at the 3′ end. The matched base between adapter sequences (R1, AGATCGGAAGAGCACACGTCTGAACTCCAGTCA; R2, AGATCGGAAGAGCGTCGTGTAGGGAAAGAGTGT) and raw data was required to be over 3 bp and mismatched base was required to be <20%. Second, fastp (v0.23.2) was used to trim low-quality reads by the sliding-window algorithm. The low-quality bases of each read were cut at the 5′ end when the base mean quality was ≥Q20 as evaluated by a sliding window (5 bp). The sequences less than 50 bp or containing ambiguous bases were removed. Third, reads aligned to the host genome by Burrows-Wheeler Aligner were removed to exclude host contamination (61). The statistics of sequencing reads of all samples are provided in Table S2.

The clean data set was produced after quality filtering of raw reads. Filtered reads were *de novo* assembled to construct the metagenome for each sample by MEGAHIT (62). The preset parameters of MEGAHIT were *k*-mers 33, 55, 77, 99, and 127. After assembly, only contigs of ≥300 bp were preserved for further analysis. minimap2 (v2.24) software was used to align quality-filtered sequences of each sample with their contig sequence sets, and the unmapped sequences were merged and reassembled (https://github.com/lh3/minimap2). The linclust mode of MMseqs2 software was used to remove redundant contigs with 95% similarity and 90% alignment area coverage (https://github.com/soedinglab/MMseqs2). The statistics of the nonredundant contigs are provided in Table S3. The lowest common ancestor taxonomy of the nonredundant genes was obtained by aligning them against the NCBI-NT database using BLASTN (expectation value threshold < 0.00001). MetaGeneMark was used to predict coding regions of metagenomic scaffolds longer than 200 bp (http://exon.gatech.edu/GeneMark/meta_gmhmmp.cgi). Coding region sequences of all samples were clustered to obtain a nonredundant gene catalog by CD-HIT, with a similarity threshold of sequence identity set to 90% (63).

Taxonomic annotation was performed using Kraken2 and Kaiju (64, 65). For samples with known reference sequences, Kraken2 software (v2.1.2 [https://github.com/DerrickWood/kraken2/wiki]) was used for taxonomic annotation by alignment with the NCBI RefSeq database at a 0.5 confidence level. The taxonomic annotation of fungal reads for all samples is provided in Table S4. When the sequence annotation rate was less than 30% by Kraken2, kaiju (v1.8.2 [https://kaiju.binf.ku.dk/]) was used instead by mapping with the NCBI NR database (the maximum number of acceptable mismatches was 5). To calculate gene abundance in each sample, minimap2 with parameters “-ax sr –sam-hit-only” was used to produce SAM files, which were then ordered and converted to BAM files using the sort function of the samtools (v1.1.4 [https://github.com/samtools/samtools]). The repeat sequences were removed by the dedup function of the bamUtil tool with default parameters (v1.0.15 [https://github.com/statgen/bamUtil]). After deduplication of these BAM files, the intersection-strict mode of the HTSeq tool was used to count the number of reads aligned to gene sequences and contig sequences and calculate the gene abundance at the different taxonomic levels (v0.12.3 [https://github.com/htseq/htseq]).

### Statistical analysis.

Student’s *t* test was used to compare alpha diversity between the no-HTN and HTN groups using Prism (GraphPad Software). Permutational multivariate analysis of variance was used to analyze principal-coordinate analysis (PCoA) distance matrix by R software (v4.0.2). Linear discriminant analysis effect size analysis was conducted to compare abundances of all fungal clades between the no-HTN and HTN groups, using Kruskal-Wallis test to obtain the *P* value. Spearman’s correlation analysis was performed and visualized by the corrplot package in R.

### Ethics approval and consent to participate.

All protocols were approved by the Institutional Review and Ethics Board of Shanghai Ninth People’s Hospital, Shanghai Jiao Tong University School of Medicine (SH9H-2018-T66-3). Informed consent was obtained from all subjects before enrollment.

### Data availability.

Raw sequences are available in the Sequence Read Archive of NIH under BioProject number PRJNA765566.
