# Supplementary material for: Characteristics and Correlations of the Oral and Gut Fungal Microbiome with Hypertension
Source: Microbiol Spectr. 2022 Dec 8;11(1):e01956-22. doi: 10.1128/spectrum.01956-22 (PMC9927468; doi:10.1128/spectrum.01956-22)
Supplement: Supplemental file 1 — Fig. S1 to S9. Download spectrum.01956-22-s0001.pdf, PDF file, 4.7 MB [file spectrum.01956-22-s0001.pdf]

Supplementary Figures

Fig. S1

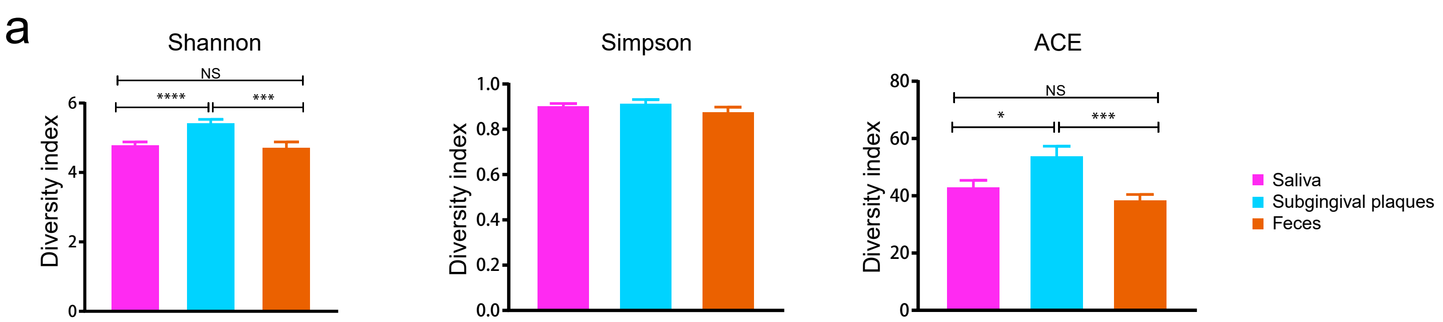

Fig. S1 The fungal diversity of oral and fecal samples in the study population. Panel correspond to the comparison of the Chao1 index in Fig 1. Shannon, Simpson, and ACE alpha diversity index of fungi in saliva, subgingival plaques, and feces. n=60 for all sample types. NS, no significance. *p < 0.05, **p < 0.01, ***p < 0.001, ****p < 0.0001.

Fig. S2

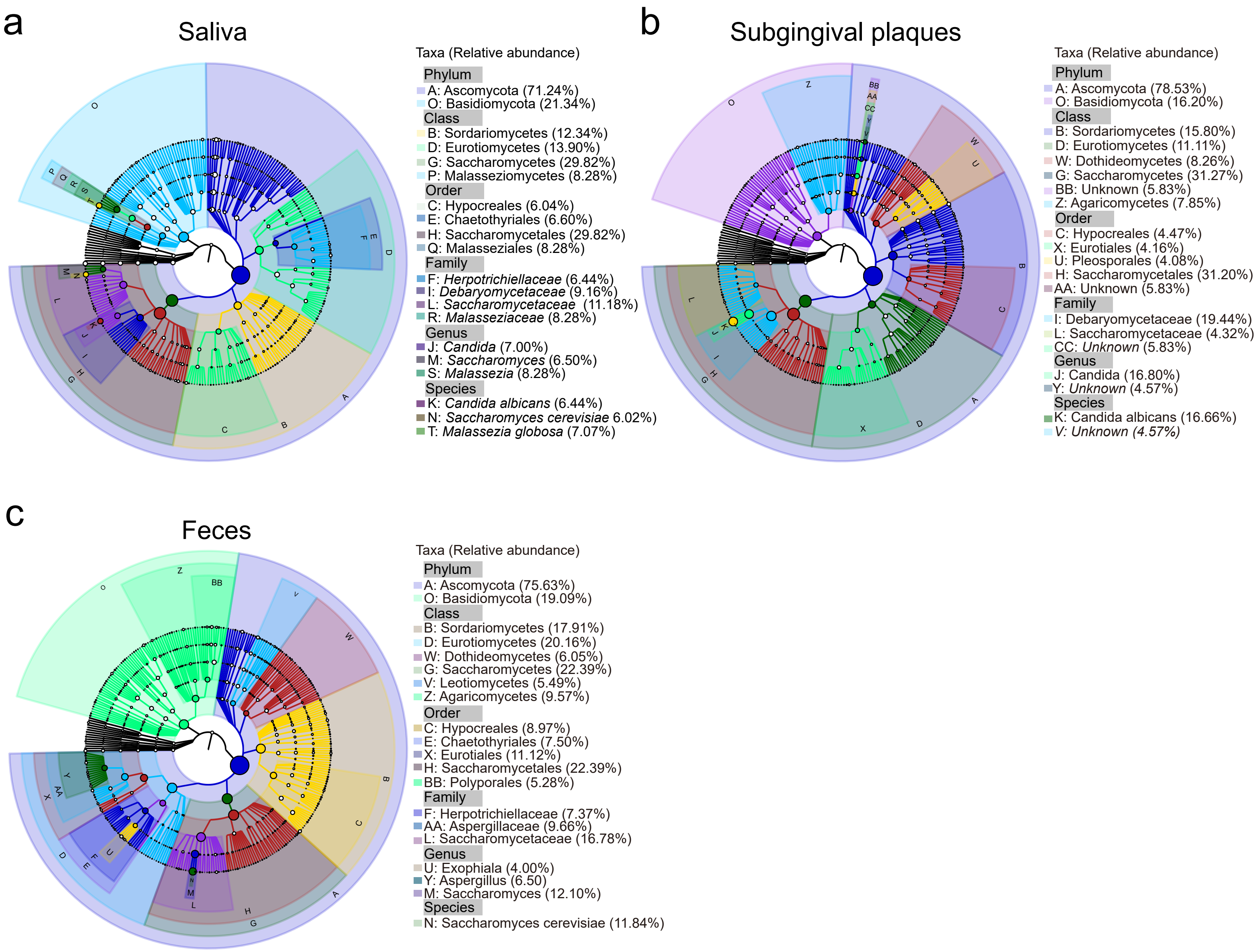

Fig. S2. Fungal phylogenetic tree of saliva, subgingival plaques, and feces. The phylogenetic trees illustrate hierarchical relationships among the top 20 fungi at different taxonomic levels from phylum to species in saliva (a), subgingival plaques (b), and feces (c). The node size represents the relative abundance of the corresponding taxon. n=60 for all sample types.

Fig. S3

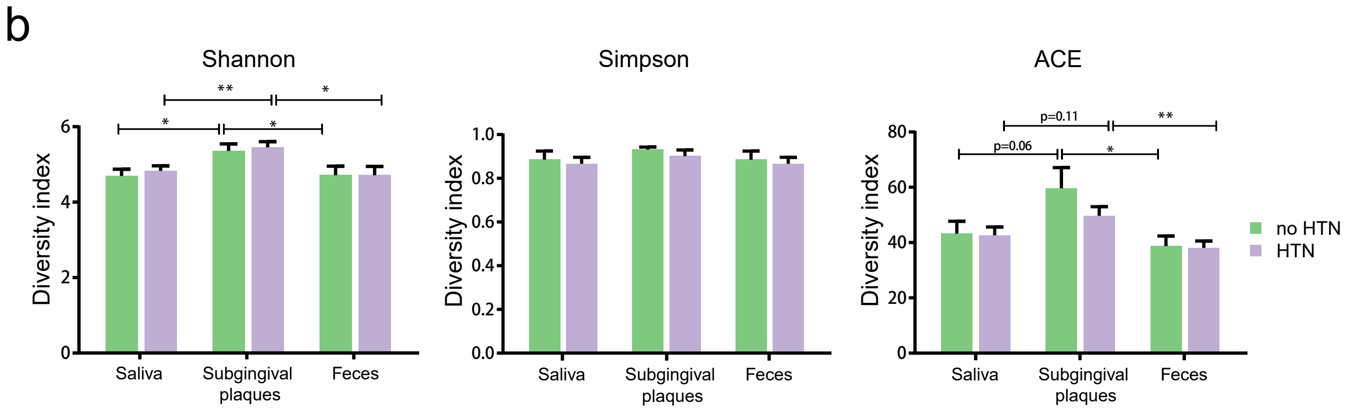

Fig. S3 The fungal diversity of oral and fecal samples between no HTN and HTN. Panel correspond to the comparison of the Chao1 index in Fig 2. Shannon, Simpson, and ACE alpha diversity index of fungi in saliva, subgingival plaques, and feces of no HTN and HTN. Student's t-test was used for statistical analysis. n=60 for all sample types. NS, no significance. *p < 0.05, **p < 0.01, ***p < 0.001, ****p < 0.0001.

Fig. S4

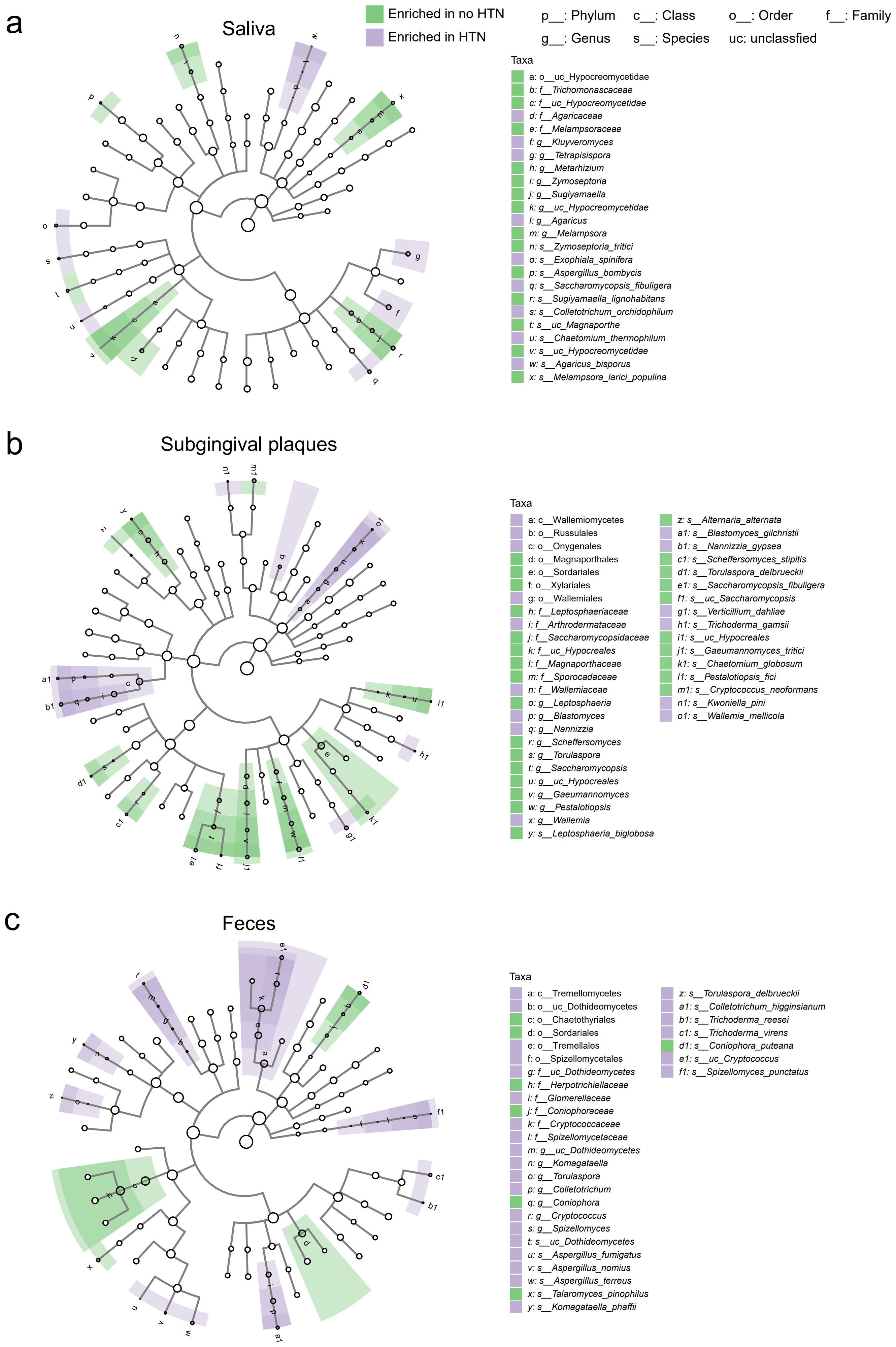

Fig. S4 Different fungal enrichment between no HTN and HTN. Cladograms of fungal microbiota in saliva (a), subgingival plaques (b), and feces (c) based on LEfSe.

Fig. S5

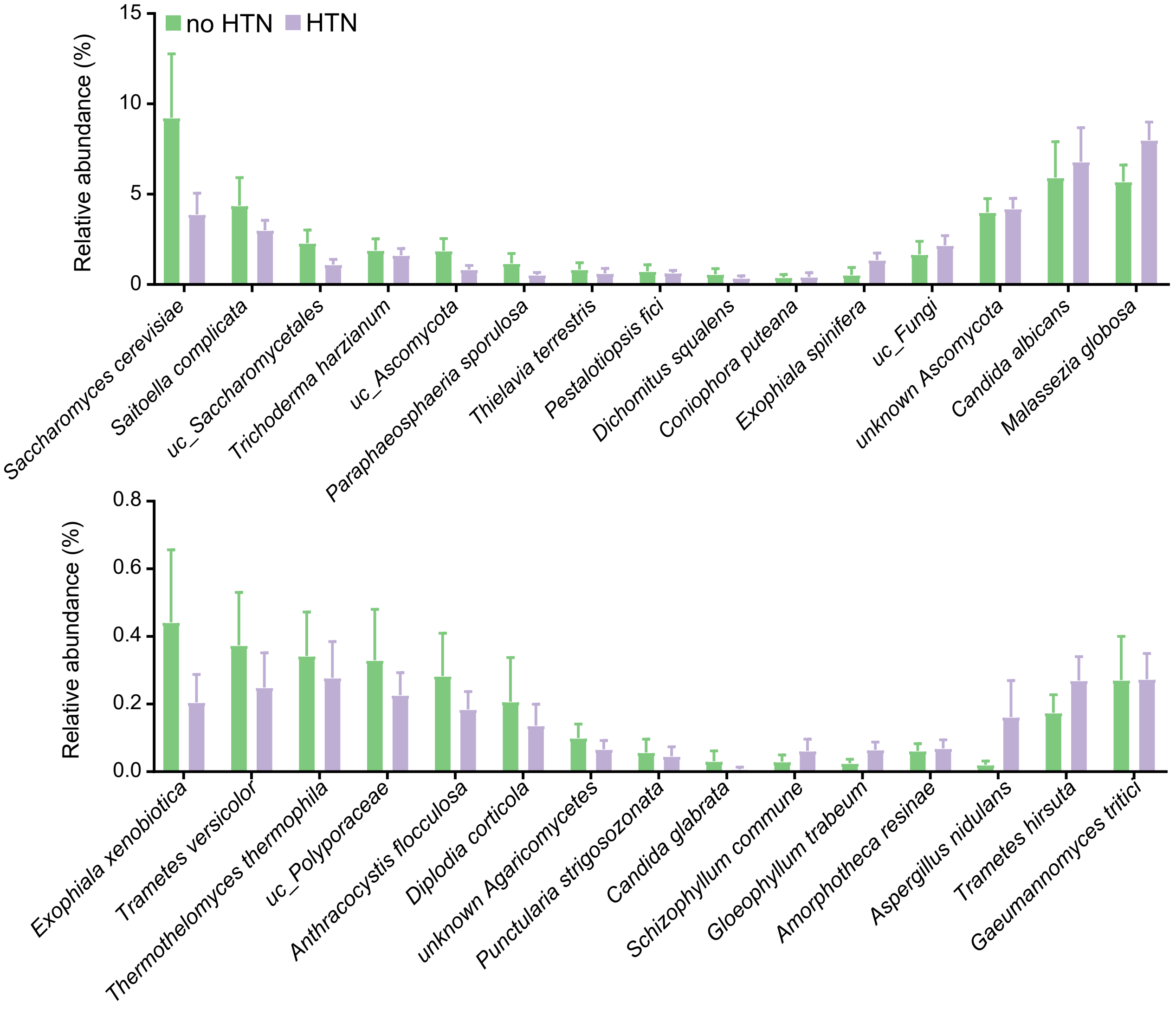

Fig. S5 Relative abundances of the oral-gut shared fungal species in saliva of no HTN and HTN. Displayed are the top 30 shared fungal species (from Fig.6A). n=24:36 (no HTN : HTN) for all sample types.

Fig. S6

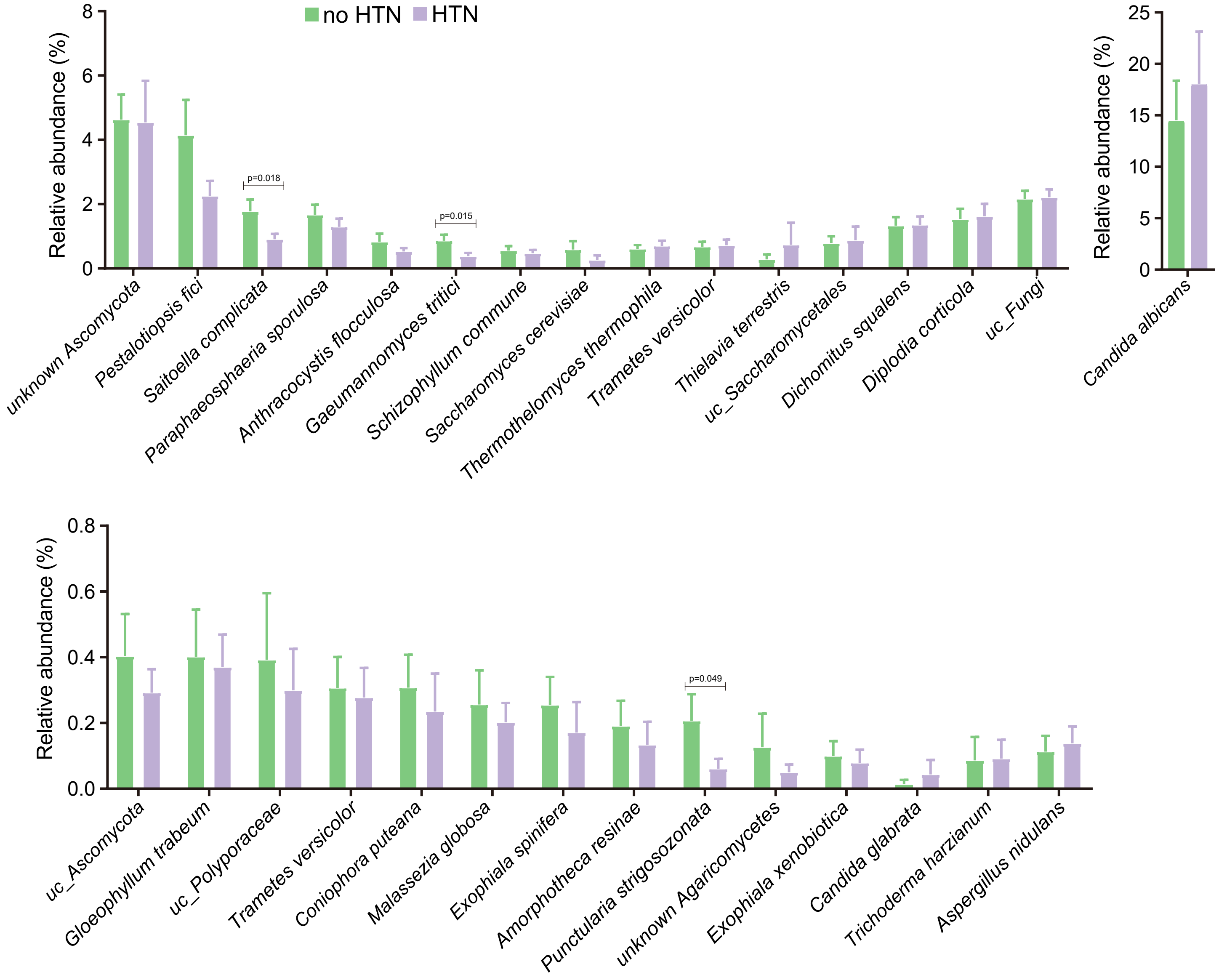

Fig. S6 Relative abundances of the oral-gut shared fungal species in subgingival plaques of no HTN and HTN. Displayed are the top 30 shared fungal species (from Fig.6A). n=24:36 (no HTN : HTN) for all sample types.

Fig. S7

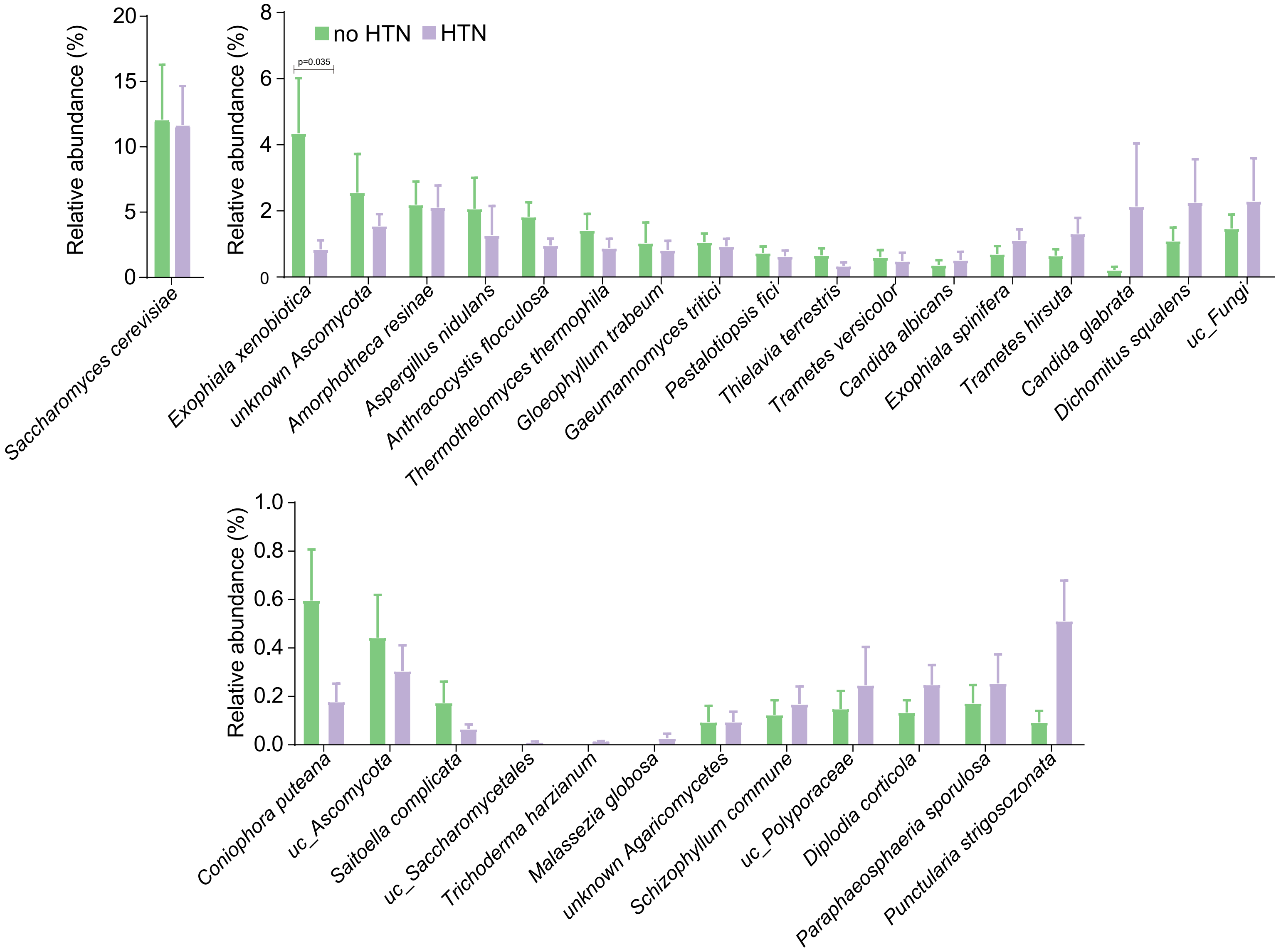

Fig. S7 Relative abundances of the oral-gut shared fungal species in feces of no HTN and HTN. Displayed are the top 30 shared fungal species (from Fig.6A). n=24:36 (no HTN : HTN) for all sample types.

Fig. S8

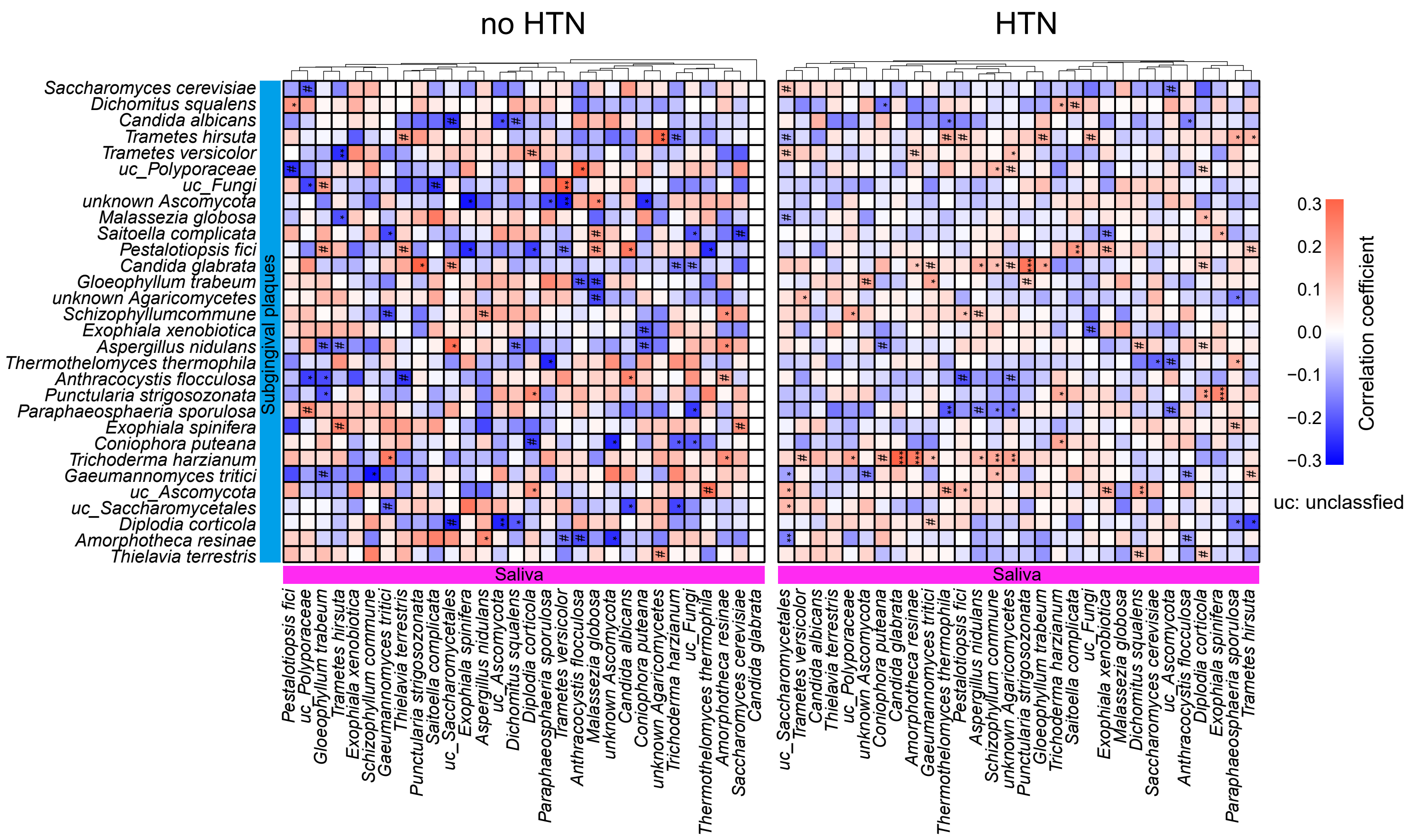

Fig. S8 Effects of HTN on associations between salivary and subgingival fungal microbiota. Heatmaps of Spearman's correlation coefficients between relative abundances of the top 30 shared fungal species (from Fig.6A) in saliva and those in subgingival plaques in no HTN and HTN participants. n=24:36 (no HTN : HTN) for both sample types. $^{\#}$p(FDR) < 0.1, *p(FDR) < 0.05, **p(FDR) < 0.01, ***p(FDR) < 0.001.

Fig. S9

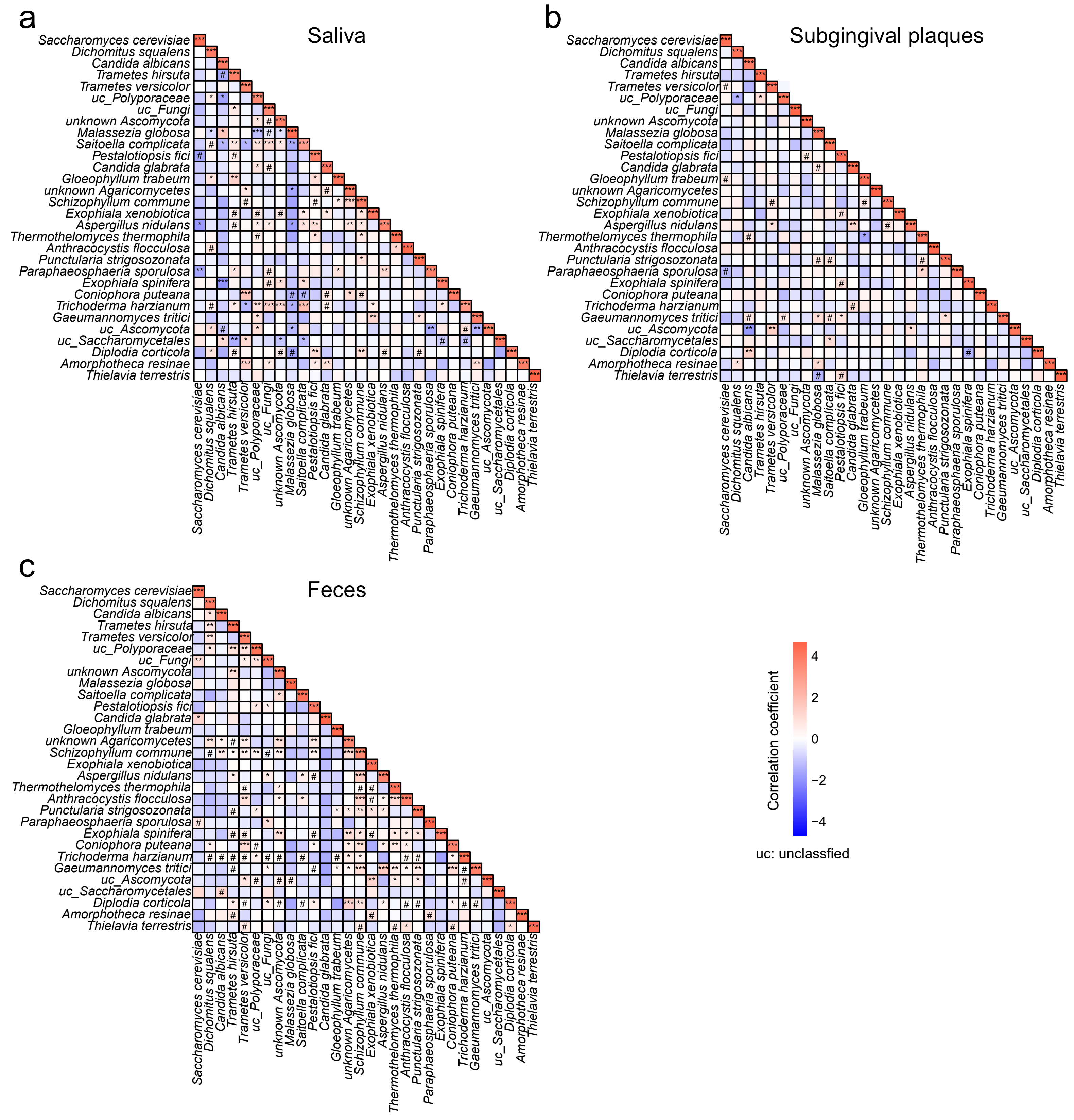

Fig. S9 Correlations among oral-gut shared fungal species within saliva, subgingival plaques, and feces. Heatmaps of Spearman's correlation coefficients among relative abundances of the top 30 shared fungal species (from Fig.6A) within saliva (a), subgingival plaques (b), and feces (c). n=60 for all sample types. $^{\#}$p(FDR) < 0.1, *p(FDR) < 0.05, **p(FDR) < 0.01, ***p(FDR) < 0.001.
